# VIM-2–producing *Pseudomonas putida*, Buenos Aires

**DOI:** 10.3201/eid1304.061083

**Published:** 2007-04

**Authors:** Marisa Almuzara, Marcela Radice, Natalia de Gárate, Alejandra Kossman, Arabela Cuirolo, Gisela Santella, Angela Famiglietti, Gabriel Gutkind, Varolos Vay

**Affiliations:** * Universidad de Buenos Aires, Buenos Aires, Argentina

**Keywords:** Carbapenem resistance, Pseudomonas putida, VIM-2, carbapenemasaes, letter

**To the Editor:** *Pseudomonas putida* (0.03% of isolates from the culture collection of the Argentina Association of Microbiology, www.aam.org.ar) infections are mainly reported in immunocompromised patients, such as newborns, neutropenic patients, and cancer patients. They are usually susceptible to extended-spectrum cephalosporins, aminoglycosides, fluoroquinolones, and carbapenems. However, isolates have been identified that produce acquired metallo-β-lactamases (MBLs) and are resistant to most β-lactams, including carbapenems.

Two multidrug-resistant *P*. *putida* isolates were obtained from clinical samples at the Sanatorio Mater Dei in Buenos Aires. One isolate was obtained in March 2005 from a urine specimen of a 76-year-old woman with a urinary tract infection who was using a urethral catheter. The second isolate was obtained in May 2005 from a tracheal aspirate of a 67-year-old man with nosocomial pneumonia.

Bacteria were identified by using conventional biochemical tests and the API 20NE System (API, bioMérieux, Lyon, France). Susceptibility tests were performed according to standard procedures. Both isolates were resistant to imipenem and meropenem (MICs >32 μg/mL) but were susceptible to amikacin and colistin. Susceptibility data are shown in the Table.

**Table:** Antimicrobial drug susceptibility profiles of 2 *bla*_VIM-2_-carrying *Pseudomonas putida* isolates, Argentina

Drug	MIC (μg/mL)	
	Isolate 1	Isolate 2
Imipenem	32	64
Meropenem	64	64
Ertapenem	128	128
Piperacillin	64	64
Piperacillin-tazobactam	32	32
Ceftazidime	128	128
Cefepime	32	32
Aztreonam	64	64
Amikacin	4	4
Gentamicin	16	16
Ciprofloxacin	>64	>64
Gatifloxacin	>64	>64
Levofloxacin	>64	>64
Moxifloxacin	>64	>64
Doxycycline	64	64
Colistin	2	2

Screening for MBLs was performed by using a double-disk diffusion method. Disks containing 1 μmol/L EDTA (metal chelator) were placed on Mueller-Hinton agar plates containing the 2 isolates. Disks containing carbapenem were placed 15 mm from disks containing EDTA. An increase in the inhibition zone of the disk containing drug near the disk containing EDTA was observed for both isolates, which suggested the presence of MBLs.

PCR amplification of *imp* and *vim* genes was conducted by using primers based on conserved regions of the *imp* and *vim* genes (*bla*IMP-F: 5′-GAAGGCGTTTATGTTCATACTT-3′, *bla*IMP-R: 5′-GTTTGCCTTACCATATTTGGA-3′, *bla*VIMG-F: 5′-GGTGTTTGGTCGCATATC-3′, and *bla*VIMG-R 5′-TGGGCCATTCAGCCAGATC-3′) and heat-extracted DNA as template. Reactions were performed in a T-gradient instrument (Biometra, Göttingen, Germany) with the following reaction conditions: 1 cycle at 95°C for 5 min, 52°C for 15 min, and 72°C for 6 min, followed by 30 cycles at 95°C for 1 min, 52°C for 1 min, and 72°C for 1 min, and a final reaction at 72°C for 20 min. Amplified fragments were sequenced on both strands by using an ABI Prism DNA 3700 (Applied Biosystems, Foster City, CA, USA), and nucleotide sequences were compared by using BLAST (National Center for Biotechnology Information, Bethesda, MD, USA, http://www.ncbi.nlm.nih.gov/Tools/). Nucleotide sequences were completely homologous to the *vim-2* coding gene.

Two repetitive-element–based PCR (rep-PCR) assays (ERIC-PCR and REP-PCR) with primers REP-1 (5′-IGCGCCGICATCAGGC-3′), REP-2 (5′-CGTCTTATCAGGCCTAC-3′), ERIC-1 (5′-CACTTAGGGGTCCTCAATGTA-3′), and ERIC-2 (5′-AAGTAAGTGACTGGGGTGAGCG-3′) were used to characterize isolates. PCR conditions were 94°C for 2 min, 30 cycles at 94°C for 30 s, 50°C for 1 min, and 72°C for 4 min, and a final reaction at 72°C for 7 min. Banding patterns were visually analyzed after electrophoresis of samples. Variations in band intensity were not considered to indicate genetic differences. Banding patterns obtained by REP-PCR and ERIC-PCR assays were identical in both isolates (data not shown).

Among the MBLs acquired by *P*. *putida*, IMP-1 was reported by Senda et al. in Japan in 1996 (*1*) and later reported in Taiwan and Japan (*2*). IMP-12 was the first IMP MBL described in *P*. *putida* in Europe (*3*). VIM-1 in *P*. *putida* was first reported in Europe (*4*), and VIM-2 in *P*. *putida* was first reported in Taiwan, Republic of Korea, Japan, and France (*5**,**6*). Our isolates were resistant to aztreonam (MIC 64 μg/mL). However, carbapenem-susceptible *P*. *putida* had low levels of susceptibility because the MIC_50_ was only 1 dilution below the current breakpoint (*7**,**8*). Aztreonam resistance could not be transferred by conjugation between IMP-1–producing (aztreonam-resistant) *P*. *putida* and *P*. *aeruginosa* (*2*) and is not associated with a transposon carrying blaVIM-2 (*6*). No evidence of extended-spectrum β-lactamases was detected in our isolates by classic synergy assays with clavulanate plus aztreonam, ceftazidime, or cefotaxime. VIM-6–producing *P*. *putida* isolates from Singapore (*9*) were more resistant to aztreonam (MIC >128 μg/mL), ceftazidime, and cefepime (MIC >256 μg/mL).

Detection of *bla*_VIM-2_ in *Pseudomonas* in South America was initially reported by the SENTRY Antimicrobial Surveillance Program (*10*) and included 1 *P*. *fluorescens* isolate in Chile and 3 *P*. *aeruginosa* isolates in Venezuela. To the best of our knowledge, our report is the first of VIM-2 in *P*. *putida* in Latin America. VIM-2–producing *P*. *putida*, which were originally restricted to East Asia and only very recently found in France, may represent an emerging pathogen or function as reservoirs for resistance because of their widespread presence in the hospital environment.
